# How Stable Are Temporal EMG Parameters in Rowing? A Seven-Day Test–Retest Reliability Study Using Wearable sEMG

**DOI:** 10.3390/s26154914

**Published:** 2026-08-04

**Authors:** Simone Kresevic, Eleonora Vignandel, Miriam Martini, Davide Arreghini, Manuela Deodato, Alex Buoite Stella, Miloš Ajčević

**Affiliations:** 1 Department of Engineering and Architecture, University of Trieste, Via Valerio 10, 34127 Trieste, Italy; simone.kresevic@dia.units.it (S.K.); eleonora.vignandel@studenti.units.it (E.V.); 2 Department of Medicine, Surgery and Health Sciences, University of Trieste, Via Pascoli 31, 34141 Trieste, Italy; miriam.martini@phd.units.it (M.M.); davide.arreghini@studenti.units.it (D.A.); mdeodato@units.it (M.D.); abuoitestella@units.it (A.B.S.)

**Keywords:** surface electromyography, wearable sensors, sEMG signal processing, rowing ergometry, muscle activation timing, test–retest reliability, neuromuscular monitoring

## Abstract

**Highlights:**

**What are the main findings?**

- Muscle activation onset showed excellent test–retest reliability across all seven muscles (ICC = 0.943–0.995), whereas active duration was poor-to-good (ICC = 0.077–0.814) and peak position poor-to-excellent (ICC = 0.114–0.948), defining a clear reliability hierarchy among temporal sEMG parameters during 2000 m rowing.
- Between-session ensemble-waveform similarity was high for every athlete across all muscles (Pearson r = 0.832–0.972; cosine similarity = 0.924–0.983), confirming that the individual neuromuscular activation fingerprint of the mean stroke cycle is reproducible over a seven-day interval.

**What are the implications of the main findings?**

- Activation timing parameters are reliable for longitudinal monitoring of neuromuscular timing in competitive rowing using wireless wearable sEMG.
- The reproducible U-shaped within-trial activation pattern provides a stable metrological baseline for detecting pacing strategy- or fatigue-driven changes across training cycles and rehabilitation programs.

**Abstract:**

Surface electromyography (sEMG), increasingly delivered through wireless wearable systems, is a key non-invasive tool for the objective monitoring of muscle activation during repetitive motor tasks. The clinical and longitudinal usefulness of wearable sEMG during rowing depends on the test–retest reliability of the parameters extracted from the signal during high-intensity, multi-muscle cyclic locomotor tasks. This study aimed to use advanced sEMG processing to quantify the between-session reliability of EMG-derived parameters (onset, offset, active duration, and peak position) across seven major muscles, to characterize the between-session similarity of ensemble-averaged activation waveforms, and to describe within-trial activation dynamics. Fifteen competitive rowers (10 males, five females; aged 14–22 years) performed two identical 2000 m all-out trials seven days apart, with sEMG recorded by a wireless wearable system. Reliability was assessed by ICC(A,1) with 95% CIs, SEM, MDC_95_, CV%, and Bland–Altman analysis. The waveform similarity of the session ensemble cycles was quantified by Pearson correlation, cosine similarity, normalized cross-correlation maximum, and normalized dynamic time warping (DTW). Within-trial dynamics were assessed across ten consecutive stroke-count windows. Onset showed excellent reliability across all seven muscles (ICC = 0.943–0.995); offset, moderate-to-excellent (0.524–0.907); peak position, poor-to-excellent (0.114–0.948); active duration, poor-to-good (0.077–0.814). Ensemble-waveform similarity between sessions was high for each athlete across all muscles (Pearson r = 0.832–0.972; cosine similarity = 0.924–0.983), confirming that the individual activation fingerprint of the mean stroke cycle is stable over a 7-day interval. Both amplitude (FMPR) and active duration revealed a reproducible U-shaped within-trial pattern. These findings highlight the potential of wearable sEMG to provide reliable, personalized insights into rowing-specific muscle activation patterns, supporting more individualized monitoring and training optimization in rowers.

## 1. Introduction

In exercise and sports physiology, as well as in rehabilitation, surface electromyography (sEMG) represents a common research tool offering the opportunity to investigate several neuromuscular parameters, including amplitudes, frequencies and additional parameters to evaluate the interaction between different muscles and fatigue during specific motor tasks [1,2]. To collect these data in more applied and realistic scenarios, for example, during trainings or competitions conducted in outdoor settings, such sEMG systems have been incorporated in textile and wearables [3], or more standard systems have been adapted to be waterproof, wireless, and often integrated with inertial measurement units (IMUs) [4]. The validity of such systems has typically been determined through the analysis of different test–retest protocols and in different motor tasks, generally reporting acceptable reliability [5,6,7].

Rowing is a sport characterized by a mixed aerobic/anaerobic metabolism, as typical performances last around 6–8 min, high power output (450–550 watts per stroke), and repetitive muscle cycles and requires a high degree of physical fitness in terms of cardiovascular endurance, muscular fitness, and maximal strength [8,9,10,11]. Several factors might affect sEMG analysis during dynamic endurance exercise, such as in rowing, including the length of the test, and despite a degree of standardization, skin impedance, perspiration, and electrode displacement, as well as subjective fatigue perception, represent potential factors that might affect data recording and subsequent analysis, representing a matter of debate [12].

In rowing, the reliability of sEMG amplitudes over repeated 2000 m sessions is generally “acceptable”, although it might not be sensitive enough to detect between-session changes related to pacing strategy [13]. Although activation amplitude is commonly used [14,15], other parameters such as muscle activation times have been used to identify the key muscles and characterize their behavior during rowing [16]. However, the reliability of these parameters has only been assessed for selected muscles and during non-rowing motor tasks [17,18]. Consequently, a comprehensive characterization of the between-session reliability of temporal sEMG-derived parameters during rowing (a prerequisite for their use in activation profile tracking and neuromuscular monitoring) is still lacking.

This study aimed to use advanced sEMG processing to quantify the between-session reliability of EMG-derived parameters (onset, offset, active duration, and peak position) across seven major muscles to characterize the between-session similarity of ensemble-averaged activation waveforms, and to describe within-trial activation dynamics.

## 2. Materials and Methods

### 2.1. Participants

Fifteen competitive rowers (10 males and 5 females; age range 14–22 years) from two rowing clubs participated in the study. All athletes were affiliated with the Italian Rowing Federation and competed at the national level, with two also being members of the Italian National Team competing internationally. Based on age category, 11 athletes were grouped as Under-17, 2 as Under-19, and two as Under-23. All participants had a minimum of three years of structured rowing training and were assessed during the in-season competitive period.

The study was conducted in accordance with the Declaration of Helsinki and was approved by the Ethics Committee of University of Trieste. Written informed consent was obtained from all adult participants and from the parents or legal guardians of underage athletes prior to enrolment.

### 2.2. Experimental Protocol

Each participant completed two identical all-out testing sessions (S0 and S1) separated by exactly seven days. Both sessions were scheduled at the same time of day to control for circadian effects, and participants were instructed to refrain from intense training in the 24 h preceding each session and to maintain habitual nutritional and hydration routines.

The session structure was identical at S0 and S1 and was performed on a Concept2 Model D rowing ergometer (Concept2 Inc., Morrisville, VT, USA), with the drag factor set according to each athlete’s habitual preference and kept identical between S0 and S1 within each participant. After surface electrode placement, the protocol consisted of (i) a 25 min standardized warm-up including three submaximal race-start simulations; (ii) a 20 s all-out maximal pull; (iii) a 5 min passive recovery; and (iv) a 2000 m all-out time-trial, with stroke rate self-selected and the only feedback provided to participants being the elapsed distance. Each performance was characterized by a Performance Index (PI), defined as the ratio between the participant’s mean 2000 m power output (recorded by the ergometer) and the world-record mean power on the rowing ergometer for the corresponding age category and sex.

The 20 s all-out test was selected for two reasons. First, the use of a sport-specific maximal task for normalization rather than an isometric maximal voluntary contraction has been shown to yield more reproducible EMG values during dynamic locomotor exercise [19,20]. Second, mean mechanical power during the 20 s all-out rowing test (W_20_) has been reported as the single strongest predictor of 2000 m completion time in young rowers [21]. The W_20_–t_2000_ relationship therefore enables verification of effort maximality at both sessions.

### 2.3. Instrumentation and Data Acquisition

Bipolar sEMG was recorded from seven muscles of the dominant side: m. Vastus Lateralis (VL), m. Vastus Medialis (VM), m. Rectus Femoris (RF), m. Biceps Femoris (BF), m. Erector Spinae Longissimus at the level of L1 (LB), m. Latissimus Dorsi (LD), and m. Biceps Brachii (BB). Skin preparation (shaving, light abrasion, and alcohol cleaning) and electrode placement followed the SENIAM guidelines [22]. EMG signals were recorded with a wireless surface EMG system (Cometa Wave Plus, Cometa s.r.l., Bareggio, Italy) at a sampling frequency of 2000 Hz, with 16-bit analog-to-digital conversion and a built-in differential amplifier (CMRR > 110 dB).

Whole-body kinematics were captured by a GoPro Hero 10 action camera (GoPro Inc., San Mateo, CA, USA), recording at 120 fps and 1080p resolution from a fixed lateral position framing the full sagittal plane of the rower. The camera was synchronized with the EMG acquisition through the proprietary EMGandMotionTools software v8.11 (Cometa s.r.l., Bareggio, Italy).

### 2.4. Signal Processing

Raw EMG signals were processed in MATLAB R2025b (The MathWorks, Natick, MA, USA). The processing pipeline applied to each muscle and each trial was (i) band-pass filtering with a 4th-order zero-phase Butterworth filter (cut-off frequencies 20 and 450 Hz); (ii) computation of the linear envelope by root-mean-square (RMS) with a 100 ms sliding window; and (iii) amplitude normalization, in which each muscle’s envelope from the 2000 m trial was divided by the mean RMS amplitude computed over the entire 20 s all-out reference trial of the same session. Normalized amplitude is expressed as a fraction of the mean activation level reached during the 20 s maximal pull.

Individual stroke cycles were segmented from catch to catch, where the catch corresponds to the position of maximal forward reach (peak hip and knee flexion). Catches were identified frame-by-frame from the synchronized video using the MediaPipe Pose framework [23,24], by tracking the hip and knee landmarks and detecting local extrema in the resulting joint-angle time series. Catch timestamps were exported and used as cycle delimiters on the EMG envelope. All strokes of the 2000 m trial were retained for analysis except for the first and last 10 strokes, which were discarded to remove kinematic inconsistencies at the start and end of the trial associated with the start- and end-spurt.

Each stroke-segmented envelope was then time-normalized to 0–100% of the cycle by cubic-spline interpolation onto a fixed grid of 1001 points (10 samples per 1% of the cycle). For each muscle, all time-normalized strokes within a session were ensemble-averaged into a single representative cycle per participant per session, yielding two ensemble cycles (S0 and S1) per muscle per participant. Subsequent parameter extraction was performed on these ensemble-averaged cycles.

### 2.5. Parameter Extraction

From each ensemble-averaged cycle, four temporal parameters were extracted, all expressed as a percentage of cycle duration:

- *Onset (%)*: the time at which the normalized EMG envelope first crossed the 15% activation threshold;
- *Offset (%)*: the time at which the envelope returned below the 15% threshold after the activation peak;
- *Active duration (%)*: the cumulative time during which the envelope exceeded a 50% activation threshold, used as an estimator of the high-activation window of the muscle;
- *Peak position (%)*: the time at which the envelope reached its maximum value within the cycle.

These thresholds were selected a priori based on a preliminary inspection of the signal envelopes and practical considerations. A low, fixed fraction of the reference amplitude (15%) was chosen to detect onset and offset because, during dynamic locomotor tasks, baseline activity is elevated relative to resting conditions, and a low percentage-of-reference threshold provides a robust criterion for separating genuine activation bursts from residual low-level activity. A higher threshold (50%) was adopted for active duration to isolate the functionally meaningful, high-activation portion of the drive phase, which is less contaminated by tonic, low-amplitude activity than estimates obtained with lower thresholds and for which no universally accepted threshold standard exists [25].

To characterize the within-trial evolution of muscle activation, each 2000 m trial was additionally divided into ten consecutive stroke-count windows, each containing 10% of the strokes of the trial (0–10%, 10–20%, …, 90–100% of strokes). Within each window, all strokes were ensemble-averaged on the time-normalized cycle, and two window-specific parameters were extracted: (i) the factor of mean power reference (FMPR), computed as the mean RMS amplitude of the window’s ensemble cycle; and (ii) the active duration (50% threshold) computed on the window’s ensemble cycle.

### 2.6. Statistical Analysis

All statistical analyses were performed in Python v3.11 using the libraries pandas v2.2, NumPy v2.4, SciPy v1.15, and pingouin v0.5.

For each of the 28 “muscles × parameter” combinations, the paired values (S0, S1) were extracted. Where data were unusable for a given muscle in one of the two sessions (electrode detachment during the trial), listwise exclusion was applied for that combination only, resulting in n = 14 for LB and n = 13 for LD. All other muscles retained the full sample size of n = 15.

Test–retest reliability was quantified using the two-way random-effects, absolute-agreement, single-measurement intraclass correlation coefficient, ICC(A,1), reported with two-sided 95% confidence intervals (Equation (1)). The absolute-agreement model was preferred over the consistency model because it incorporates systematic between-session shifts, providing a conservative measure appropriate for instrument validation. ICC values were classified following Koo and Li [26] as Poor (<0.50), Moderate (0.50–0.74), Good (0.75–0.89), or Excellent (≥0.90).

(1) $$ICC\left(A,1\right)=\frac{{MS}_{R}-{MS}_{E}}{{MS}_{R}+\left(k-1\right){MS}_{E}+\frac{k(MS_{C}-MS_{E})}{n}},$$

where ${MS}_{R}$ is the mean square for rows (between subjects), ${MS}_{C}$ the mean square for columns (between sessions), ${MS}_{E}$ the residual mean square error, k is the number of sessions (2), and n the number of participants.

Absolute measurement error was characterized by standard error of measurement (SEM, Equation (3)) and minimal detectable change at 95% confidence (MDC_95_, Equation (4)). Relative variability was assessed by the coefficient of variation of paired differences (CV%, Equation (5)).

(2) $${SD}_{pooled}=\sqrt{\frac{{SD}_{0}^{2}+{SD}_{1}^{2}}{2}},$$

(3) $$SEM={SD}_{pooled}\cdot \sqrt{1-ICC},$$

(4) $${MDC}_{95}=1.96\cdot \sqrt{2}\cdot SEM,$$

(5) $$CV\%=\frac{{SD}_{diff}}{{mean}_{all}\cdot \sqrt{2}}\cdot 100,$$

Systematic bias and agreement between sessions were assessed by Bland–Altman analysis, computing the mean bias of the paired differences (d_i_ = S1_i_ − S0_i_), the 95% limits of agreement (bias ± 1.96 × ${SD}_{diff}$), and visual inspection for proportional bias and outliers. The statistical significance of the bias was tested by a paired-samples *t*-test (H_0_: mean(d_i_) = 0), preceded by Shapiro–Wilk testing of the normality of the differences.

Within-trial differences across the ten stroke-count windows were tested for FMPR and active duration separately for each muscle, by means of a non-parametric repeated-measures Friedman test, given the moderate sample size and the presence of skewed distributions for some “muscle × window” combinations.

Statistical significance was set at α = 0.05 (two-sided).

### 2.7. Waveform Similarity Analysis

To assess the between-session reproducibility of the individual activation pattern, the ensemble-averaged EMG cycle of each participant was compared between sessions at the subject level. For each participant j and each muscle, the S0 and S1 ensemble cycles (each a vector $s\in R^{1001}$ spanning 0–100% of the normalized stroke cycle) were treated as a matched pair and their similarity quantified by four complementary metrics (Equations (6)–(9)).

The Pearson correlation coefficient (r, Equation (6)) measures the linear shape agreement between the two cycles after mean-centering, independently of amplitude scaling.

(6) $$r_{j}=\frac{\sum_{k}\left(s_{j,k}^{0}-{\bar{s}}_{j}^{0})\cdot (s_{j,k}^{1}-{\bar{s}}_{j}^{1}\right)}{\sqrt{\sum_{k}{\left(s_{j,k}^{0}-{\bar{s}}_{j}^{0}\right)}^{2}\cdot \sum_{k}{\left(s_{j,k}^{1}-{\bar{s}}_{j}^{1}\right)}^{2}}},$$

Cosine similarity (Equation (7)) measures the angle between the two cycles treated as vectors in $R^{1001}$, capturing shape agreement without mean-centring and thus retaining sensitivity to the baseline activation level.

(7) $$\cos\theta_{j}=\frac{s_{j}^{0}\cdot s_{j}^{1}}{\left\|s_{j}^{0}\right\|\cdot \left\|s_{j}^{1}\right\|},$$

The normalized cross-correlation maximum (XCorr, Equation (8)) extends Pearson r by permitting a rigid temporal shift τ between cycles, thereby isolating shape dissimilarity from any constant phase offset.

(8) $${XCorr}_{j}=\underset{\tau }{\max}\frac{\sum_{k}{\tilde{s}}_{j,k}^{0}\cdot {\tilde{s}}_{j,k+\tau }^{1}}{\left\|{\tilde{s}}_{j}^{0}\right\|\cdot \left\|{\tilde{s}}_{j}^{1}\right\|},$$

The normalized Dynamic Time Warping (DTW, Equation (9)) measures the distance between the two cycles divided by the length of the optimal warping path $\left|P_{j}^{*}\right|$, accommodating non-linear local temporal deformations (lower values indicate greater similarity).

(9) $${DTW}_{j}=\frac{d_{DTW}(s_{j}^{0},s_{j}^{1})}{\left|P_{j}^{*}\right|},$$

where k indexes the 1001 time points of the normalized cycle and ${\tilde{s}}_{j}=s_{j}-{\bar{s}}_{j}$ denotes the mean-centred cycle of participant j. Each participant thus serves as their own reference, consistent with the paired design adopted throughout. Group results are reported as mean ± SD across the n valid participants (n = 13–15 depending on muscle). All waveform similarity metrics were computed in a Python v3.11 environment using the libraries SciPy and NumPy.

## 3. Results

### 3.1. Participants and Performance

The sample of 15 rowers and the corresponding performance values at S0 and S1 are summarized in Table 1. Mean 2000 m completion time was 7:18.8 ± 0:44.3 (mm:ss) (range 6:10.3–8:29.2) at S0 and 7:17.6 ± 0:44.0 (range 6:04.4–8:29.7) at S1. Mean 2000 m power was 280.8 ± 85.3 W at S0 and 283.1 ± 87.3 W at S1. Mean W_20_ was 506.1 ± 168.6 W at S0 and 508.7 ± 170.7 W at S1. Within-session W_20_ was inversely correlated with t_2000_ at S0 (r = −0.981, R^2^ = 0.962, *p* < 0.0001) and S1 (r = −0.962, R^2^ = 0.926, *p* < 0.0001). The Performance Index was 0.61 ± 0.10 at S0 (range 0.50–0.78) and 0.61 ± 0.10 at S1 (range 0.51–0.82).

### 3.2. Reliability Analysis

**Figure 1:**
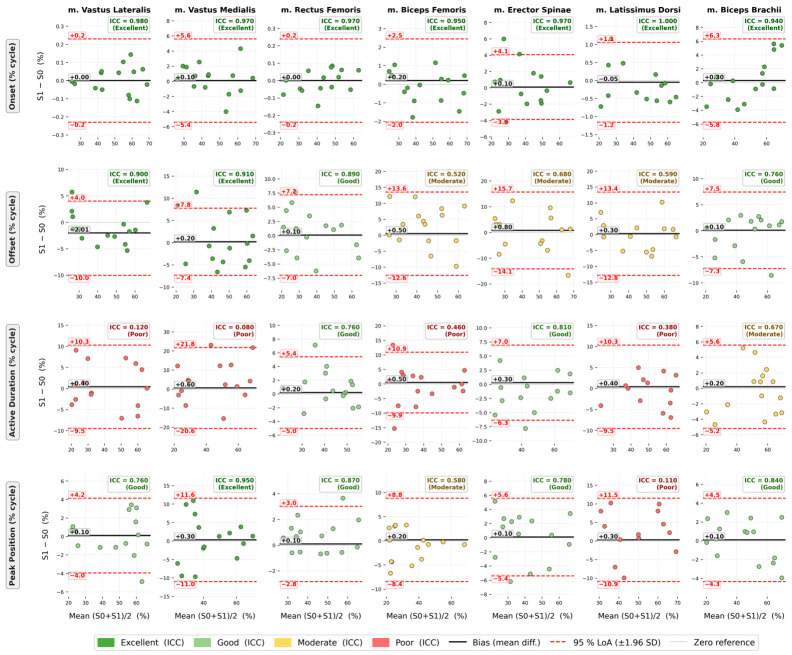
Bland–Altman analysis of between-session agreement for four temporal EMG parameters (onset, offset, active duration, peak position) across seven muscles. Each panel shows individual paired differences (S1 − S0) plotted against the session mean. ICC(A,1) classification is color-coded: Excellent (green), Good (light green), Moderate (orange), Poor (red).

Across the 28 “muscle × parameter” combinations, ICC(A,1) classification was excellent for ten (35.7%), good for eight (28.6%), moderate for five (17.9%), and poor for five (17.9%) combinations. A total of 18/28 (64.3%) combinations exceeded ICC ≥ 0.75 and 23/28 (82.1%) exceeded ICC ≥ 0.50. Systematic bias was absent in 27/28 combinations (paired *t*-test, all *p* > 0.05), with the sole exception of offset for VL (bias = −2.01%, *p* = 0.007); this result did not survive Bonferroni correction for the 28 comparisons (threshold *p* < 0.0018). Full ICC(A,1) values and complete numerical results (including SEM, MDC_95_, CV % and bias) are reported in Table 2. Bland–Altman plots for all combinations are shown in Figure 1.

Onset reliability was excellent for all seven muscles, with ICC(A,1) ranging from 0.943 (BB) to 0.995 (LD). No combination showed significant systematic bias (all *p* ≥ 0.088). Offset reliability was excellent for VL (ICC = 0.904) and VM (0.907), good for RF (0.889) and BB (0.758), and moderate for BF (0.524), LB (0.679), and LD (0.592). Active duration showed good reliability for LB (ICC = 0.814) and RF (0.764), moderate for BB (0.665), and poor for VL (0.118), VM (0.077), BF (0.455), and LD (0.377). Peak position reliability was excellent for VM (ICC = 0.948), good for RF (0.871), BB (0.835), LB (0.780), and VL (0.756), moderate for BF (0.575), and poor for LD (0.114).

### 3.3. Waveform Similarity Analysis

**Figure 2:**
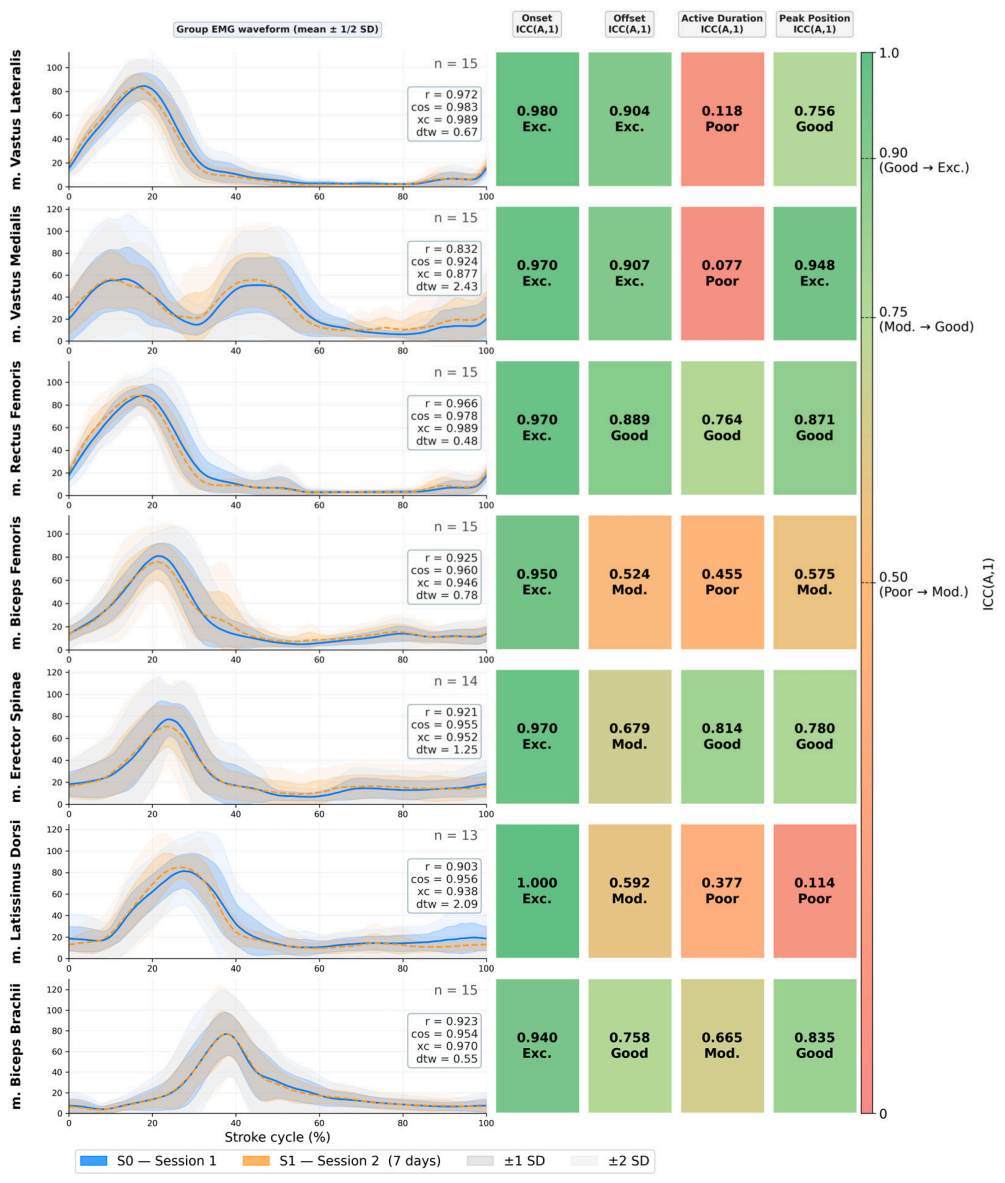
Ensemble-averaged EMG waveforms and between-session reliability of temporal parameters across seven muscles. **Left** panels show group mean ± 2 SD for Session 0 (S0, blue) and Session 1 (S1, red), expressed as normalized amplitude (fraction of 20 s maximal reference) over the time-normalized stroke cycle (0–100%). Waveform similarity metrics (Pearson r, cosine similarity, normalized cross-correlation xc, and normalized DTW) are reported for each muscle. **Right** panel summarizes ICC(A,1) values for each parameter × muscle combination, color-coded by reliability class.

Between-session similarity of the ensemble-averaged EMG cycle was high across all seven muscles (Table 3; Figure 2).

Mean Pearson r ranged from 0.832 ± 0.143 (VM) to 0.972 ± 0.030 (VL). Six of seven muscles exceeded r ≥ 0.90 on average; only VM (0.832) fell below this threshold. Inter-subject variability was highest for VM (SD = 0.143) and lowest for VL (SD = 0.030) and RF (SD = 0.039). Cosine similarity was higher than the corresponding Pearson r for all seven muscles. Mean values ranged from 0.924 ± 0.060 (VM) to 0.983 ± 0.018 (VL). The largest difference relative to Pearson r was observed for VM (Δ = +0.092), LD (Δ = +0.053), BF (Δ = +0.035), and LB (Δ = +0.034); the smallest for VL (Δ = +0.011) and RF (Δ = +0.012). Cross correlation ranged from 0.877 ± 0.102 (VM) to 0.989 ± 0.015 (RF) and 0.989 ± 0.017 (VL). Values exceeded 0.93 for all muscles except VM (0.877). DTW was lowest for RF (0.48 ± 0.33), BB (0.55 ± 0.27), and VL (0.67 ± 0.73), and markedly higher for LD (2.09 ± 1.75) and LB (2.30 ± 1.57). Standard deviations exceeded the mean for VL and LB, indicating right-skewed distributions with high-leverage individual values.

### 3.4. Within-Trial Dynamics Across Stroke-Count Windows

**Figure 3:**
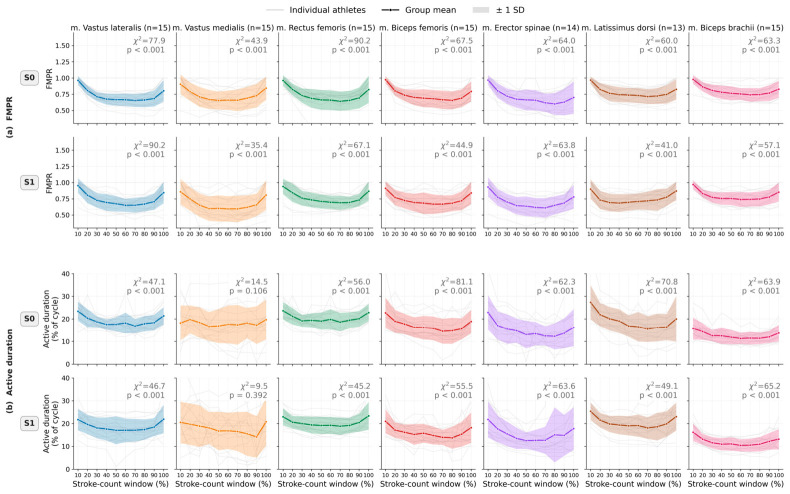
Within-trial activation dynamics across ten consecutive stroke-count windows during the 2000 m trial, shown for Session 0 (S0) and Session 1 (S1). Colors denote individual muscles. (**a**) Factor of mean power reference (FMPR): mean RMS amplitude of each window’s ensemble cycle, normalized to the 20 s maximal reference trial. (**b**) Active duration (50% threshold): cumulative proportion of the stroke cycle during which normalized EMG exceeded 50%. Shaded bands represent ± 1 SD across participants. χ^2^ and *p*-values from Friedman tests are reported for each muscle × session combination.

The within-trial pattern of FMPR and active duration across the ten stroke-count windows of the 2000 m trial is shown in Figure 3 for both sessions. FMPR described the variation in the mean activation level (Figure 3a), whereas high intensity active duration described the variation in the window length (Figure 3b).

*FMPR.* A clear U-shaped pattern emerged in all seven muscles, with the highest mean values observed during the first 0–10% window (drive phase of the initial strokes) and a relative recovery during the last window (end-spurt preparation), separated by a sustained “valley” between approximately 40% and 80% of the strokes. At S0, mean FMPR at the 0–10% window ranged from 1.10 (BB) to 1.18 (BF) of the 20 s reference, and reached troughs between 0.72 (LB, 70–80% window) and 0.84 (LD/BB, 60–70% window). The Friedman test detected a significant effect of stroke-count window on FMPR for all seven muscles at both sessions (S0: χ^2^(9) = 43.9–90.2, all *p* < 0.001; S1: χ^2^(9) = 35.4–90.2, all *p* < 0.001).

*Active duration.* A similar but attenuated U-shape was observed in six of seven muscles at both sessions, with mean values at S0 ranging from approximately 11–15% of the cycle in the central windows and rising to 16–27% in the boundary windows. The Friedman test was significant for VL, RF, BF, LB, LD, and BB at both sessions (S0: χ^2^(9) = 47.1–81.1, all *p* < 0.001; S1: χ^2^(9) = 45.2–65.2, all *p* < 0.001). VM was the sole muscle showing no significant effect of stroke-count window on active duration at either session (S0: χ^2^(9) = 14.5, *p* = 0.106; S1: χ^2^(9) = 9.5, *p* = 0.392), indicating that the high-activation window length of VM remained stable throughout the 2000 m trial despite the documented amplitude variation.

## 4. Discussion

To the best of the authors’ knowledge, this is the first study to provide a comprehensive evaluation of the test–retest reliability of temporal sEMG parameters in rowing. A previous study reported that, for most of the investigated muscles, the repeatability of the rectified sEMG amplitudes was “large” to “very large” [13]. In the current study, muscle activation “onset” and “offset” showed moderate to excellent ICC values, suggesting good between-session reliability; however, the quantification of active duration was poor in three of the seven muscles, particularly for the m. Vastus Lateralis, m. Vastus Medialis and m. Latissimus Dorsi, the last of which was the only muscle also characterized by poor test–retest reliability for the “peak position” parameter. Interestingly, m. Vastus Medialis and m. Latissimus Dorsi were also among the muscles characterized by a lower ICC in previous work [13].

The lower reliability of active duration and peak position in specific muscles likely reflects both methodological and physiological factors. Active duration depends on two crossings of a 50% amplitude threshold, so in muscles whose envelope is broad and plateau-like around this level (as in the VL and VM), small between-session amplitude fluctuations shift the crossing points substantially, inflating duration variability even when the overall waveform shape is highly reproducible. Peak position, derived from a single argmax, is similarly unstable on flat or multimodal profiles, where the global maximum can jump between adjacent peaks across sessions, and this particularly affects LD. LD also presents an additional, electrode-related source of error. As a broad, sheet-like muscle overlain by skin that moves considerably during the rowing stroke, it is particularly prone to movement artefact and cross-talk, consistent with its highest DTW distance and the electrode detachment events that reduced its effective sample size. By contrast, activation onset is identified once at the steep rising edge of the activation burst and is largely immune to these effects, which explains its excellent reliability across all muscles. These estimates should be read alongside their uncertainty. With 13–15 participants, several ICC confidence intervals were wide and occasionally spanned more than one reliability category, so the point-estimate classifications should not be interpreted in isolation. Practically, parameters with poor reliability are not advisable as stand-alone monitoring markers, whereas activation onset is well suited to this purpose. For each parameter, the MDC_95_ in Table 2 defines the change that a between-session difference must exceed to be considered genuine rather than a measurement error, and we recommend using these values when tracking individual athletes.

In the sport setting, activation “onset” and “offset” parameters can be used to define the pattern of muscle activation during specific gestures [27,28,29], which could be affected by factors such as fatigue [30,31], injuries and rehabilitation [32,33]. Similarly, the duration of muscle activation has also been used to describe neuromuscular patterns in sports, where it has been related with gesture effectiveness and athletes’ expertise [34]. Finally, “time-of-peak”, which in this study was reported as the movement cycle where the peak occurred (“peak position”), has previously been used to describe the muscle activation sequence during motor tasks [35,36].

In rowing, these parameters have been used to describe muscle activation sequence and characteristics on an indoor rowing machine [16]. According to this study, the plantar flexors, as well as the ventral and dorsal thigh muscles, were predominantly active during the drive phase (i.e., when knees are extended) from just before the catch to just before the finish, whereas the tibialis anterior was recruited during the recovery phase (i.e., when knees are flexed, without significant body side differences). In more detail, m. Rectus Femoris and m. Biceps Femoris were respectively active for the longest and briefest periods during the rowing gesture [16]. From an applied perspective, neuromuscular activation patterns have been suggested to be associated with better performance and biomechanical efficiency; for example, different activation profiles of lower and upper trunk flexors suggest different purposes within a rowing cycle. Indeed, increased m. rectus abdominis activation during the recovery phase may be detrimental to performance, whereas greater arm muscle activation during the early recovery phase predicts higher performance [36].

Taken together, previous studies suggest a potential role of sEMG-derived parameters to better describe sporting gestures, and this might also support the development of new training, prevention, and rehabilitation strategies in rowing. Nevertheless, some sources of bias should be considered, as several factors might affect sEMG analysis during dynamic endurance exercise, and considering the different sensitivities of different sEMG indexes, it is recommended that they be combined with different indicators for a comprehensive analysis [37]. The reliability of these indexes has only been partially reported specifically during rowing; the results of the present study suggest that for the most commonly assessed muscles of the lower limb, trunk, and upper limb, the evaluation of muscle activation times can be considered acceptable overall during indoor rowing.

Beyond their reproducibility, the waveform-similarity metrics offer a methodological advantage over the discrete temporal parameters because they integrate information across the whole normalized cycle. They are far more robust to the local amplitude fluctuations that destabilize threshold- and instant-based measures. This explains the dissociation seen for the m. Vastus Medialis, whose activation pattern is stable yet whose threshold-based duration is fragile, and means the metrics recover activation information the discrete parameters can miss. This robustness also has a practical use. Because each athlete’s ensemble profile was highly reproducible over seven days, the individual waveform can serve as a personalized baseline, and a marked drop in similarity would flag a meaningful change in the activation pattern rather than normal day-to-day variation. This is useful for monitoring technique, fatigue-related changes in coordination, or recovery during rehabilitation. Indeed, systematic waveform changes could also be related to performance gains or losses, although this would require coupling the similarity metrics with performance outcomes in a longitudinal design [34,36,38].

The reproducible U-shaped within-trial profile of both FMPR and active duration mirrors the pacing strategy typically adopted during 2000 m rowing. There is a high-activation explosive start to overcome flywheel inertia and establish race pace, a sustained central “valley” as the athlete settles into a metabolically sustainable steady state with lower recruitment demands, and a terminal rise reflecting the end-spurt. The terminal increase likely reflects the compensatory recruitment of higher-threshold motor units to maintain target power output as peripheral fatigue progressively reduces the force-generating capacity of the already-active pool. Active duration follows the same U-shaped pattern because higher-amplitude bursts naturally spend a larger fraction of the cycle above the 50% threshold. Importantly, this profile was statistically significant at both sessions for all muscles except VM, indicating that it constitutes a stable, individually reproducible neuromuscular pacing template rather than a random fluctuation.

However, some limitations should be acknowledged. The test was performed on an indoor rowing ergometer, and data were collected during a self-selected stroke-rate 2000 m all-out time-trial. Although the duration and intensity were chosen to match typical training and competition conditions and thereby improve the ecological validity of the study, this remains a potential source of bias; moreover, it is not possible to generalize these findings to on-water rowing, as most studies only applied sEMG analysis indoors [39,40]. Several sample-related limitations should also be acknowledged. The sample was small (n = 15) and only comprised young competitive rowers, so the estimates may not generalize to adult elite, recreational, or clinical populations, in which technique and recruitment strategies differ (e.g., technique refinement in elite athletes reduces inter-individual variability, while clinical populations present altered recruitment). The reliability hierarchy reported here should be confirmed in other cohorts. The cohort was also heterogeneous in age category and sex; because the ICC reflects the ratio of between-subject to total variance, this heterogeneity may have inflated some coefficients, so the ICCs should be regarded as sample-specific. Studies with more homogeneous cohorts would be expected to yield narrower and possibly lower ICC values. Additionally, electrode detachment during the maximal effort reduced the sample to n = 14 (erector spinae) and n = 13 (latissimus dorsi), lowering statistical power and widening the confidence intervals for these muscles and, together with the latissimus dorsi’s artefact susceptibility, partly explaining its weaker reliability. Reinforced electrode fixation and larger, stratified samples are recommended for future work to confirm the present findings and to enable between-group comparisons (e.g., by age category, maturational stage, sex, or training experience). Finally, the temporal parameters were derived using fixed amplitude thresholds (15% for onset/offset; 50% for active duration). Because no universally accepted threshold standard exists [25], the chosen values may influence the resulting estimates, potentially limiting the generalizability of these findings to other methods. The waveform-similarity analysis, which is threshold-independent, is not subject to this limitation and may therefore offer a more robust approach to characterizing sEMG timing across studies.

## 5. Conclusions

This study demonstrated that parameters extracted via the processing of wearable surface electromyography signals provide reproducible temporal information during high-intensity rowing. These findings define a clear reliability hierarchy among temporal sEMG-derived variables during cyclic rowing exercise. Beyond isolated temporal parameters, the ensemble-averaged activation waveform showed high between-session similarity across all muscles, supporting the concept that each athlete presents a stable and reproducible neuromuscular activation fingerprint during the rowing stroke cycle. Moreover, the consistent U-shaped within-trial behavior observed for both activation amplitude and active duration suggests the presence of a reproducible neuromuscular pacing strategy throughout the 2000 m effort.

These findings highlight the potential of wearable sEMG to provide reliable, personalized insights into rowing-specific muscle activation patterns, supporting more individualized monitoring and training optimization in rowers. Wearable sEMG could serve as a valuable longitudinal monitoring tool for evaluating activation timing characteristics during training, fatigue monitoring, and rehabilitation.

## Figures and Tables

**Table 1:** Participant characteristics and performance at Session 0 (S0) and Session 1 (S1). PI: Performance Index (ratio of participant mean 2000 m power to world-record mean power for the corresponding age category and sex).

	Males (n = 10)	Females (n = 5)	Total (n = 15)
Body height (cm)	183.2 ± 4.3	166.4 ± 6.3	178.0 ± 9.5
Body mass (kg)	82.3 ± 9.0	62.0 ± 8.8	75.3 ± 13.1
Training experience (yr)	5.4 ± 1.78	5.4 ± 1.1	5.4 ± 1.5
Category (u17/u19/u23)	7/1/2	4/1/0	11/2/2
S0			
PI (%)	61.7 ± 10.5%	58.9 ± 8.7%	60.8 ± 9.7%
time_2000_ (mm:ss)	6:53.0 ± 0:28.5	8:07.6 ± 0:23.6	7:18.8 ± 0:44.3
Mean Power_2000m_ (W)	323.4 ± 69.8	195.5 ± 29.4	280.8 ± 85.3
Mean Power_20s_ (W)	600.5 ± 97.1	317.4 ± 105.8	506.1 ± 168.6
S1			
PI (%)	62.3 ± 10%	58.9 ± 7.2%	61.2 ± 9.0%
time_2000_ (mm:ss)	6:52.2 ± 00:28.1	8:06.8 ± 00:19.9	7:17.6 ± 0:44.0
Mean Power_2000m_ (W)	326.7 ± 72.3	195.7 ± 24.3	283.1 ± 87.3
Mean Power_20s_ (W)	603.9 ± 103.8	318.4 ± 174.9	508.7 ± 170.7

**Table 2:** Test–retest reliability of four temporal EMG parameters across seven muscles. Abbreviations: ICC: intraclass correlation coefficient, absolute-agreement, two-way random-effects model ICC(A,1); CI: confidence interval; SEM: standard error of measurement; MDC_95_: minimal detectable change at 95% confidence; CV%: coefficient of variation of paired differences. n = 15 for all muscles except erector spinae (n = 14) and latissimus dorsi (n = 13).

Title 1	ICC (95% CI)	SEM	MDC	CV%
Onset				
m.VL	0.98 [0.94–0.99]	0.08	0.23	21.8%
m.VM	0.97 [0.92–0.99]	1.99	5.52	36.9%
m.RF	0.97 [0.90–0.99]	0.09	0.24	34.1%
m.BF	0.95 [0.85–0.98]	0.81	2.25	36.2%
m.LB	0.97 [0.92–0.99]	1.43	3.97	23.1%
m.LD	1.00 [0.98–1.00]	0.40	1.11	8.5%
m.BB	0.94 [0.83–0.98]	2.18	6.05	9.7%
Offset				
m.VL	0.90 [0.57–0.97]	2.17	6.03	5.4%
m.VM	0.91 [0.75–0.97]	2.73	7.57	5.2%
m.RF	0.89 [0.71–0.96]	2.56	7.11	7.6%
m.BF	0.52 [0.04–0.81]	4.72	13.07	13.2%
m.LB	0.68 [0.26–0.88]	5.39	14.94	13.9%
m.LD	0.59 [0.06–0.86]	4.74	13.15	10.8%
m.BB	0.76 [0.42–0.91]	2.65	7.36	4.8%
Active duration (50% threshold)				
m.VL	0.12 [−0.45–0.59]	3.57	9.90	19.8%
m.VM	0.08 [−0.49–0.57]	7.66	21.23	43.0%
m.RF	0.76 [0.43–0.91]	1.89	5.23	9.6%
m.BF	0.46 [−0.60–0.78]	3.76	10.43	23.9%
m.LB	0.81 [0.53–0.94]	2.40	6.65	16.9%
m.LD	0.38 [−0.21–0.76]	3.42	9.94	17.8%
m.BB	0.67 [0.26–0.87]	1.46	5.39	15.2%
Peak				
m.VL	0.76 [0.43–0.91]	1.46	4.05	8.3%
m.VM	0.95 [0.85–0.98]	4.07	11.29	15.2%
m.RF	0.87 [0.66–0.96]	1.06	2.93	5.8%
m.BF	0.58 [0.10–0.83]	3.12	8.63	13.9%
m.LB	0.78 [0.45–0.92]	1.98	5.50	8.0%
m.LD	0.11 [−0.30–0.57]	4.05	11.24	14.7%
m.BB	0.84 [0.57–0.94]	1.60	4.44	4.4%

**Table 3:** Between-session waveform similarity of the ensemble-averaged EMG cycle across seven muscles. Values are mean ± SD across participants.

Muscle	n	Pearson r	Cosine Similarity	Cross Corr.	DTW
m. Vastus Lateralis	15	0.972 ± 0.03	0.983 ± 0.018	0.989 ± 0.017	0.666 ± 0.730
m. Vastus Medialis	15	0.832 ± 0.143	0.924 ± 0.060	0.877 ± 0.102	2.429 ± 1.570
m. Rectus Femoris	15	0.966 ± 0.039	0.978 ± 0.023	0.989 ± 0.015	0.478 ± 0.331
m. Biceps Femoris	15	0.925 ± 0.065	0.960 ± 0.029	0.946 ± 0.068	0.776 ± 0.534
m. Erector Spinae	14	0.921 ± 0.062	0.955 ± 0.035	0.952 ± 0.054	1.245 ± 1.299
m. Latissimus Dorsi	13	0.903 ± 0.128	0.956 ± 0.049	0.938 ± 0.083	2.087 ± 1.752
m. Biceps Brachii	15	0.923 ± 0.059	0.954 ± 0.036	0.970 ± 0.025	0.553 ± 0.268

## Data Availability

Data are unavailable due to privacy restrictions. Access to the data may be granted on reasonable request for the purpose of scientific collaboration, subject to approval by the corresponding author.

## Abbreviations

The following abbreviations are used in this manuscript:

VL	m. Vastus Lateralis
VM	m. Vastus Medialis
RF	m. Rectus Femoris
BF	m. Biceps Femoris
LB	m. Erector Spinae
LD	m. Latissimus Dorsi
BB	m. Biceps Brachii
sEMG	Surface electromyography
EMG	Electromyography
ICC	Intraclass correlation coefficient
CI	Confidence interval
SEM	Standard error of measurement
MDC_95_	Minimal detectable change at 95% confidence
CV%	Coefficient of variation of paired differences
DTW	Dynamic time warping
FMPR	Factor of mean power reference
IMU	Inertial measurement units
S0	Testing session 0
S1	Testing session 1
PI	Performance index
W_20_	Mean mechanical power during the 20 s all-out rowing test
t_2000_	2000 m completion time
RMS	Root-mean-square
MS	Mean square
SD	Standard deviation

## References

[1] Vigotsky A.D., Halperin I., Lehman G.J., Trajano G.S., Vieira T.M. (2018). Interpreting signal amplitudes in surface electromyography studies in sport and rehabilitation sciences. Front. Physiol..

[2] Martini M., Cargnel A., Raffini A., Mazzari L., Ajcevic M., Accardo A., Canton G., Deodato M., Buoitestella A., Murena L. (2025). Effects of swimming fatigue on neuromuscular parameters in young swimmers with unilateral shoulder pain. J. Sports Med. Phys. Fit..

[3] Medagedara M.H., Ranasinghe A., Lalitharatne T.D., Gopura R.A.R.C., Nandasiri G.K. (2024). Advancements in Textile-Based sEMG Sensors for Muscle Fatigue Detection: A Journey from Material Evolution to Technological Integration. ACS Sens..

[4] Chang K.M., Liu S.H., Wu X.H. (2012). A wireless sEMG recording system and its application to muscle fatigue detection. Sensors.

[5] Lynn S.K., Watkins C.M., Wong M.A., Balfany K., Feeney D.F. (2018). Validity and reliability of surface electromyography measurements from a wearable athlete performance system. J. Sports Sci. Med..

[6] Amin A.B., Asabre E., Sahay A., Razaghi S., Noh Y. Feasibility Testing of Wearable Device for Musculoskeletal Monitoring during Aquatic Therapy and Rehabilitation. Proceedings of the Annual International Conference of the IEEE Engineering in Medicine and Biology Society, EMBS.

[7] Bangaru S.S., Wang C., Aghazadeh F. (2020). Data quality and reliability assessment of wearable emg and IMU sensor for construction activity recognition. Sensors.

[8] Penichet-Tomás A., Pueo B. (2017). Performance conditional factors in rowing. Retos.

[9] Penichet-Tomas A., Pueo B., Jimenez-Olmedo J.M. (2016). Relationship between experience and training characteristics with performance in non-olympic rowing modalities. J. Phys. Educ. Sport.

[10] Cerasola D., Bellafiore M., Cataldo A., Zangla D., Bianco A., Proia P., Traina M., Palma A., Capranica L. (2020). Predicting the 2000-m Rowing Ergometer Performance from Anthropometric, Maximal Oxygen Uptake and 60-s Mean Power Variables in National Level Young Rowers. J. Hum. Kinet..

[11] Thiele D., Prieske O., Chaabene H., Granacher U. (2020). Effects of strength training on physical fitness and sport-specific performance in recreational, sub-elite, and elite rowers: A systematic review with meta-analysis. J. Sports Sci..

[12] Daniel N., Małachowski J., Sybilski K., Błażkiewicz M. (2026). Muscle Fatigue in Dynamic Movement: Limitations and Challenges, Experimental Design, and New Research Horizons. Bioengineering.

[13] Gee T.I., Mulloy F., Gibbon K.C., Stone M.R., Thompson K.G. (2023). Reliability of electromyography during 2000 m rowing ergometry. Sport Sci. Health.

[14] Czajkowska U., Świątek-Najwer E., Jankowski L. (2023). Analysis of muscle activity during rowing stroke phases. Acta Bioeng. Biomech..

[15] Vinther A., Alkjaer T., Kanstrup I.-L., Zerahn B., Ekdahl C., Jensen K., Holsgaard-Larsen A., Aagaard P. (2011). Neuromuscular activity and force production during slide-based and stationary ergometer rowing. Br. J. Sports Med..

[16] Vieira T.M., Cerone G.L., Stocchi C., Lalli M., Andrews B., Gazzoni M. (2020). Timing and modulation of activity in the lower limb muscles during indoor rowing: What are the key muscles to target in FES-rowing protocols?. Sensors.

[17] Gupta A., Mudie K.L., Clothier P.J. (2014). The reliability of determining the onset of medial gastrocnemius muscle activity during a stretch-shorten-cycle action. J. Electromyogr. Kinesiol..

[18] Carvalho C.R., Fernández J.M., Del-Ama A.J., Oliveira Barroso F., Moreno J.C. (2023). Review of electromyography onset detection methods for real-time control of robotic exoskeletons. J. Neuroeng. Rehabil..

[19] Albertus-Kajee Y., Tucker R., Derman W., Lamberts R.P., Lambert M.I. (2011). Alternative methods of normalising EMG during running. J. Electromyogr. Kinesiol..

[20] Albertus-Kajee Y., Tucker R., Derman W., Lambert M. (2010). Alternative methods of normalising EMG during cycling. J. Electromyogr. Kinesiol..

[21] Cataldo A., Cerasola D., Russo G., Zangla D., Traina M. (2015). Mean power during 20 sec all-out test to predict 2000 m rowing ergometer performance in national level young rowers. J. Sports Med. Phys. Fit..

[22] Hermens H.J., Freriks B., Disselhorst-Klug C., Rau G. (2000). Development of recommendations for SEMG sensors and sensor placement procedures. J. Electromyogr. Kinesiol..

[23] Lugaresi C., Tang J., Nash H., McClanahan C., Uboweja E., Hays M., Zhang F., Chang C.-L., Yong M.G., Lee J. (2019). MediaPipe: A Framework for Building Perception Pipelines. arXiv.

[24] Bazarevsky V., Grishchenko I., Raveendran K., Zhu T., Zhang F., Grundmann M. (2020). BlazePose: On-device Real-time Body Pose tracking. arXiv.

[25] Tenan M.S., Tweedell A.J., Haynes C.A. (2017). Analysis of statistical and standard algorithms for detecting muscle onset with surface electromyography. PLoS ONE.

[26] Koo T.K., Li M.Y. (2016). A Guideline of Selecting and Reporting Intraclass Correlation Coefficients for Reliability Research. J. Chiropr. Med..

[27] Cochrane D.J., Barnes M.J. (2015). Muscle activation and onset times of hip extensors during various loads of a closed kinetic chain exercise. Res. Sports Med..

[28] Vasudevan J.M., Logan A., Shultz R., Koval J.J., Roh E.Y., Fredericson M. (2016). Comparison of Muscle Onset Activation Sequences between a Golf or Tennis Swing and Common Training Exercises Using Surface Electromyography: A Pilot Study. J. Sports Med..

[29] Ortega-Cebrián S., Girabent-Farrés M., Whiteley R., Navarro R., Monné-Guasch L., Bagur-Calafat C. (2019). Shoulder muscle onset timing during clinical assessment movements is the same in elite handball players as non-athletes: Implications for clinical assessment. Phys. Ther. Sport.

[30] Pouliquen C., Nicolas G., Bideau B., Bideau N. (2021). Impact of Power Output on Muscle Activation and 3D Kinematics During an Incremental Test to Exhaustion in Professional Cyclists. Front. Sports Act. Living.

[31] Chen S.F., Wang Y., Bing F., Zhang M. (2023). The effects of alteration in muscle activation on the iliotibial band during an exhaustive run. BMC Sports Sci. Med. Rehabil..

[32] Russo F., Ghislieri M., Baldazzi A., Borzuola R., Rum L., Bergamini E., Agostini V. (2025). Delayed muscle onset in athletes after anterior cruciate ligament reconstruction during unplanned change-of-directions. Gait Posture.

[33] Dingenen B., Peeraer L., Deschamps K., Fieuws S., Janssens L., Staes F. (2015). Muscle-activation onset times with shoes and foot orthoses in participants with chronic ankle instability. J. Athl. Train..

[34] Pakosz P., Domaszewski P., Konieczny M., Bączkowicz D. (2021). Muscle activation time and free-throw effectiveness in basketball. Sci. Rep..

[35] Spudić D., Smajla D., Burnard M.D., Šarabon N. (2021). Muscle activation sequence in flywheel squats. Int. J. Environ. Res. Public Health.

[36] Pitto L., Ertel G.N., Simon F.R., Gauchard G.C., Mornieux G. (2024). Influence of Neuromuscular Activity and Technical Determinants on Scull Rowing Performance. Appl. Sci..

[37] Li J., Rizna F., Zheng X. (2023). Surface electromyography changes of male athletes during 2000 m rowing ergometer test. J. Mens. Health.

[38] Stefanovic F., Ramanarayanan S., Karkera N.U., Mujumdar R., Sivaswaamy Mohana P., Hostler D. (2022). Rate of change in longitudinal EMG indicates time course of an individual’s neuromuscular adaptation in resistance-based muscle training. Front. Rehabil. Sci..

[39] Fleming N., Donne B., Mahony N. (2014). A comparison of electromyography and stroke kinematics during ergometer and on-water rowing. J. Sports Sci..

[40] Schwensow D., Hohmuth R., Malberg H., Schmidt M. Investigation of muscle fatigue during on-water rowing using surface EMG. Proceedings of the Annual International Conference of the IEEE Engineering in Medicine and Biology Society, EMBS.

